# Expression of Toll‐like receptors in nasal epithelium in allergic rhinitis

**DOI:** 10.1111/apm.12408

**Published:** 2015-06-08

**Authors:** Jutta Renkonen, Sanna Toppila‐Salmi, Sakari Joenväärä, Pirkko Mattila, Ville Parviainen, Jaana Hagström, Caj Haglund, Mikko Lehtonen, Risto Renkonen

**Affiliations:** ^1^ Transplantation LaboratoryHaartman InstituteUniversity of HelsinkiHelsinkiFinland; ^2^ Department of AllergyUniversity of Helsinki and Helsinki University Central HospitalHelsinkiFinland; ^3^ Institute for Molecular Medicine Finland (FIMM)University of HelsinkiHelsinkiFinland; ^4^ Department of PathologyHaartman InstituteUniversity of HelsinkiHelsinkiFinland; ^5^ HUSLABUniversity of Helsinki and Helsinki University Central HospitalHelsinkiFinland; ^6^ Department of SurgeryUniversity of Helsinki and Helsinki University Central HospitalHelsinkiFinland; ^7^ Research Programs Unit, Translational Cancer BiologyUniversity of HelsinkiHelsinkiFinland; ^8^ Department of OtorhinolaryngologyTampere University HospitalTampereFinland

**Keywords:** Allergic rhinitis, challenge, epithelium, immunohistochemistry, toll‐like receptor

## Abstract

Toll‐like receptors (TLRs) are important in barrier homeostasis, but their role in airborne allergies is not fully understood. The aim was to evaluate baseline and allergen‐induced expression of TLR proteins in nasal epithelium during allergic rhinitis. Nineteen otherwise healthy non‐smoking volunteers both allergic to birch pollen and non‐allergic controls were enrolled. We took nasal biopsies before and after off‐seasonal intranasal birch pollen or diluent challenge. The expression of epithelial TLR1‐7, TLR9‐10, and MyD88 proteins was immunohistochemically evaluated from the nasal biopsies. The TLR1‐3 and TLR5‐10 mRNAs were observed by RNA‐microarray. Baseline epithelial expression of TLR proteins was wide and identical in controls and atopics. After off‐seasonal intranasal birch pollen challenge, a negative change in the expression score of TLR1 and TLR6 proteins was detected in the atopic group. TLR mRNA expression was not affected by birch pollen challenge. Nasal epithelium seems to express all known TLRs. The mechanisms by which TLR1, and TLR6 proteins could affect pollen allergen transport need further studies.

**Abbreviations**
AR: allergic rhinitis
LPS: lipopolysaccharide
MyD88: myeloid differentiation primary response 88
PRR: pattern recognition receptor
RIG‐I: retinoic acid‐inducible gene I
Th‐1: T‐helper cell 1
Th‐2: T‐helper cell 2
TIR: Toll‐interleukin 1 receptor
TIRAP: toll‐interleukin 1 receptor (TIR) domain containing adaptor protein
TLR: Toll‐like receptor
TRAM: toll‐like receptor 4 adaptor protein
TRIF: TIR‐domain‐containing adapter‐inducing interferon‐β

Birch pollen allergic rhinitis is the most common allergic disorder in the Northern Europe, with a prevalence of 15–20% 1, 2. Epigenetic and genetic modifications of innate immunity together with microbial and other environmental stimuli may predispose to airway allergy 3, 4. Many intrinsic and environmental factors facilitate the entry of airborne allergens in the respiratory mucosa 3. Epithelial cells produce mediators, which affect the recruitment and activation of more specialized immune cells and create a microenvironment where these activated immune cells may function and propagate the inflammatory processes 5.

Innate immunity by pathogen recognition is a pivotal defense system. Its aim is fast detection of pathogens from the environment when they get into contact with the organisms. At least four classes of pattern recognition receptors (PRRs) identified to date are critical for sensing microorganisms and for the subsequent stimulation of proinflammatory responses: toll‐like receptors (TLRs), nucleotide oligomerization domain‐like receptors, RIG‐I‐like receptors, and C type lectin receptors 6. Human TLRs are a large family with at least eleven members 5. TLRs sense a large diversity of pathogen‐associated molecular patterns from various intruders such as bacteria and viruses, bacterial lipopolysaccharide (LPS), lipoproteins, peptidoglycans, bacterial DNA, and double‐stranded RNA 7, 8, 9, 10. TLR1, TLR2, TLR4, TLR5, TLR6, and TLR10 are expressed on the epithelial cell surface and recognize the pathogen‐associated molecular patterns of extracellular microbes 8, 11, 12, 13. TLR3, TLR7, TLR8, and TLR9 are localized within the intracellular endolysosomal compartments and are involved in the recognition of nucleic acids. TLR3 utilizes exclusively the TRIF‐dependent pathway. The signals of other TLRs utilize the MyD88‐dependent pathways as well 14. For instance, upon recognition of LPS on the cell surface, TLR4 first induces the TIRAP/MyD88 signaling on the plasma membrane and is thereafter endocytosed. Next TRAM‐TRIF is activated in early endosomes leading to induction of type‐I interferons 7, 8, 9, 10, 11.

Under normal conditions commensal bacteria are recognized by TLRs and this recognition is essential for the maintenance of homeostasis and a state of constant controlled inflammation 15. Different mutations and experimental models, which alter the TLR functions, have demonstrated the significance of TLRs in susceptibility to infection 16, 17, 18. TLRs are also reported to be involved in the pathogenesis of a large number of inflammatory disorders, such as asthma and allergy 13, chronic rhinosinusitis 19, inflammatory bowel disease 20, atherosclerosis 21, and obesity 22. Meta‐analyses of genome‐wide studies indicate that loci in the region TLR1‐TLR6‐TLR10 might associate with atopic sensitization or reported allergy 23, 24. Der p 2, the main house dust mite allergen, has shown to mimic MD2 like the chaperone that promotes TLR4 signaling 25. Previous studies show that the following TLRs might be related with grass or birch pollen allergic rhinitis: TLR1, TLR2, TLR6, TLR7, TLR8, and TLR10 23, 26, 27, 28, 29.

We previously demonstrated that birch pollen allergic patients might have reduced immune response in their nasal epithelium 30. This could putatively lead to an active epithelial transport of birch pollen allergens detected only in patients allergic to birch pollen 31. The aim of this study was to evaluate whether the baseline epithelial expression of TLRs and MyD88 differ between healthy and birch pollen allergic subjects. Moreover, we aimed at detecting allergen‐induced early alterations in the expression of TLRs and MyD88.

## Materials and Methods

### Subjects

This study was carried out at the University of Helsinki and Tampere University Hospital in 2007‐08. It was approved by the Ethical Committee of the Hospital District of Pirkanmaa (nro. R04044), and was performed according to the Declaration of Helsinki. Written informed consent was obtained from all subjects. Nineteen subjects (nine allergic and 10 healthy) participated in the study (Table 1). The subjects were Caucasian and were either atopic with allergic rhinoconjunctivitis symptoms, or non‐atopic. The diagnosis of birch‐induced allergic rhinitis was based on a history of spring seasonal allergic rhinitis, clinical examination, and skin prick test positivity according to ARIA‐guidelines 1. Characteristics of the subject groups are shown in Table 1. Exclusion criteria were smoking, acute respiratory infection during the experiment, other diseases than allergic rhinoconjunctivitis, regular use of medication, as well as nasal endoscopic findings of moderate or severe septal deviation, nasal polyps, or mucopurulent discharge.

**Table 1:** The antibodies (all IgG) and their concentrations used in immunohistochemistry for detecting TLR 1‐7 and 9‐10 proteins. The mAb anti‐TLR8 was not commercially available when the work was performed, and thus was not used

TLR1 (H‐90):sc‐30000 Santa Cruz Biotechnology, Inc., Dallas, Texas, USA, 1:100, 2 μg/mL
TLR2 (H‐175):sc‐10739 Santa Cruz Biotechnology, Inc., 1:50, 4 μg/mL
TLR3 (H‐125):sc‐10740 Santa Cruz Biotechnology, Inc. 1:50, 4 μg/mL
TLR4 (H‐80):sc‐10741 Santa Cruz Biotechnology, Inc. 1:50, 4 μg/mL
TLR5 (IMG‐664A) Imgenex, San Diego, California, USA, 1:200, 2.5 μg/mL
TLR6 (IMG‐304A) Imgenex, 1:3000, 0.17 μg/mL
TLR7 (IMG‐581A) Imgenex, 1:300, 1.67 μg/mL
TLR9 (H‐100):sc‐25468 Santa Cruz Biotechnology, Inc. 1:100, 2 μg/mL
TLR10 (DDX0490) Dendritics, Lyon, France, 1:200, 2.5 μg/mL
MyD88 (ab2068) Abcam, Cambridge, UK, 1:800, 1.25 μg/mL

### Nasal challenge and biopsies

The subjects participated in the challenge experiment in January, which is the season with no flowering outdoor plants in Finland. The local anesthesia and biopsy techniques have been previously described 32. Briefly, the first biopsy was taken with Fokkens′ forceps from the anterior edge of the right inferior turbinate before the challenge. The challenge was performed by putting 3–5 drops of either birch pollen solution (Betula Verrucosa, Alutard SQ, 10 000 SQ‐U, ALK‐Abelló*,* H*ø*rsholm, Denmark) or diluent (ALK‐Diluent; ALK‐Abelló) on the left nasal inferior turbinate. The second biopsy was taken from the left inferior turbinate 3 min after challenge. The patients were asked not to use medication (antihistamine and/or nasal corticosteroids) for a minimum of 5 days before specimens were taken. However, all subjects reported that they had not needed to use medication for a longer time than 4 weeks before the specimens were taken.

### Symptoms scores

The patients were asked the following symptoms before and 20 min after taking the biopsies: nasal and eye itching; nasal congestion, discharge, sneezing, pain, and bleeding. The scores for itching symptoms and congestion‐discharge‐sneezing symptoms were used in this study. These two scores were determined from the asked corresponding symptoms semiquantitatively 0 (no symptoms), 1 (mild), 2 (moderate), 3 (severe).

### Immunohistochemistry and light microscope evaluation

The nasal biopsies were formalin‐fixed and paraffin‐embedded. Four μm sections of paraffin blocks were deparaffinized in xylene and rehydrated in decreasing concentration of ethanol to distilled water. Slides were pretreated in a PreTreatment module (Lab Vision Corp., Fremont, CA, USA) in Tris‐HCl (pH 8.5) for antigen retrieval and stained in an Autostainer 480 (Lab Vision Corp.). Primary antibodies are listed in Table 1. For detection of bound antibodies Dako REAL EnVision Detection system, Peroxidase/DAB+, Rabbit/Mouse (Dako, Glostrup, Denmark) was used. Slides were counterstained with hemalaun‐eosin. Mucosa of the oral and nasal cavities, placenta and pancreas served as positive controls. The specificity of immunohistochemistry was controlled by omitting the primary antibodies. Two researchers (JR and JH) scored the stained sections independently. Cases of disagreement were discussed, and a consensus score was determined for further analysis. The staining score was determined semiquantitatively from the samples: 0 (no positively stained cells); 1 (< 5–20% of the cells were positive); 2 (20–50% of the cells were positive); 3 (> 50–80% of the cells were positive); 4 (80–100% of the cells were positive). For data analyses, we used the staining scores and the delta staining scores. The delta staining score was counted in the following way: the delta staining score – staining score _postchallenge_ – staining score _prechallenge_. Inflammation in the nasal specimens was based on the semiquantitatively assessed amount of inflammatory cell (lymphocyte and polymorphonuclear leukocyte) infiltration in the mucosa, and was scored by: 0 = no inflammation, 1 = mild inflammation, 2 = moderate inflammation, 3 = strong inflammation.

### mRNA levels of TLR1‐10

For the RNA‐microarray assay, we used nasal biopsies taken from six healthy and seven allergic subjects before and 3 min after the intranasal birch or diluent challenge. For RNA isolations Rneasy Mini kit (Qiagen, Hilden, Germany) was used according to the instructions of the manufacturer. RNA integrity and quantity were measured with AgilentBioanalyser RNA 6000 Nano kit and NanoDrop spectrophotometer, respectively. The processed RNA samples were prepared and hybridized on Illumina human WG‐6 v2 chips in Finnish DNA Microarray Centre (Turku Centre for Biotechnology, Turku, Finland). Chipster analysis software was used for basic statistics and data normalization (quantile) purposes and IPA software (Ingenuity; Qiagen) comparing the groups of samples and interpreting the data, respectively 33.

### Data analysis

Statistical analysis was carried out by the PASW statistics 18.0 Statistical Software Package (SPSS Inc., Chicago, IL, USA). Data are expressed as means and as medians when specified. For comparisons, the results were analyzed by, Fisher's exact test (discrete) or Kruskal–Wallis and Mann–Whitney *U* (MWU) tests (continuous). For pair‐wise comparisons Wilcoxon test was used. Two‐tailed p‐values of < 0.05 were considered statistically significant.

## Results

### The baseline nasal epithelial expression of TLR proteins in healthy and allergic subjects

**Figure 1:**
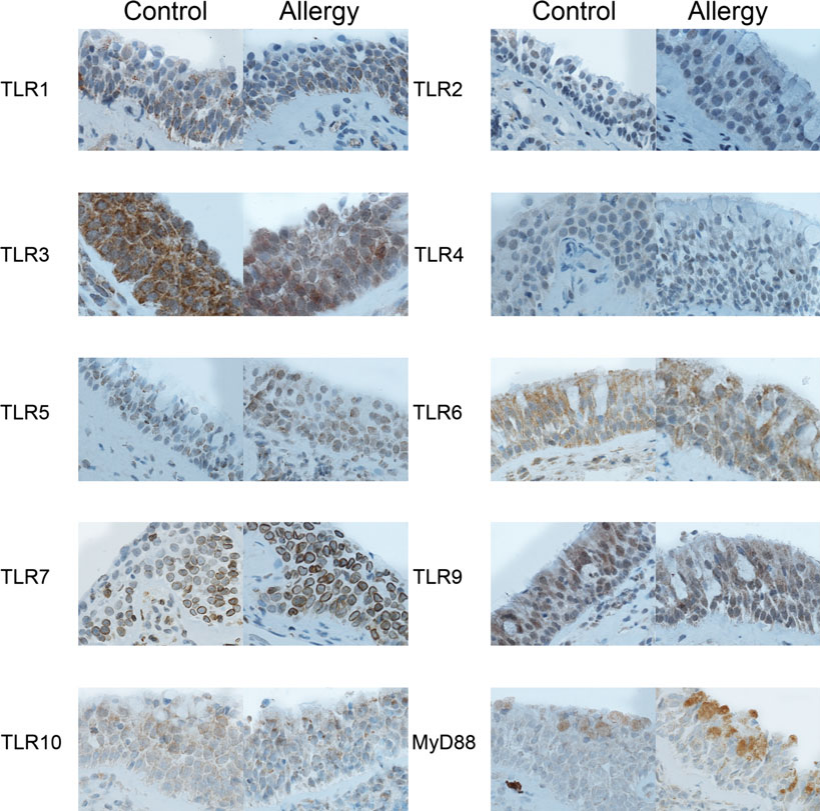
The baseline expression of the TLR1‐7 and TLR9‐10 proteins in the nasal epithelium from healthy controls (Control) and subjects with birch pollen allergic rhinitis (Allergy) during winter. Magnification ×100 in all panels, except in TLR2 A, TLR 3 Control and Allergy; and TLR6 Allergy, where magnification is ×200.

**Figure 2:**
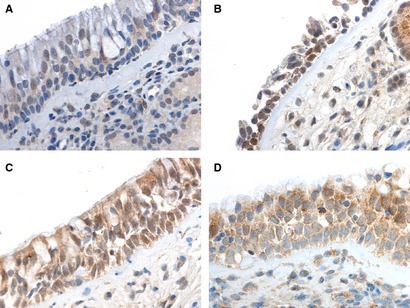
The intra‐epithelial expression of TLRs. The expression of TLR2 (A) is weak close to nuclei. The expression of TLR4 (B) is moderate and unevenly distributed in the epithelium. The expression of TLR9 (C) and TLR10 (D) is granular throughout the epithelium. Magnification is ×600 in all panels.

The atopic and control groups did not differ by age, male‐female ratio, or by number and percentage of peripheral blood eosinophils (p < 0.05, Table 2). Atopic patients had significantly increased total IgE, birch specific IgE, and timothy specific IgE levels in serum (Table 2). We detected expression of TLR 1‐7 and 9‐10 proteins in nasal epithelial layer and glandular epithelial cells (Figs 1 and 2). In addition, mild expression of TLR 3 and TLR5‐7 was detected in mucosal leukocytes and endothelial cells. In this study we focused on the epithelial expression. In winter and before challenge, median epithelial staining scores of TLR 1‐7 and TLR 9‐10 proteins did not differ between the atopic and control groups (p > 0.05, by Mann–Whitney *U*‐test, data not shown). The expression of TLR1 was moderate and granular, and mostly found in the lower half of the nasal epithelium. The expression of TLR2 was weak and found close to nuclei, whereas that of TLR3 was strong, granular and detected in all epithelial compartments. The expression of TLR4 and TLR5 was moderate and unevenly distributed in the epithelium. Interestingly, a strong expression of TLR6 was found throughout the whole nasal epithelium in both groups. A moderate TLR7 expression was mainly detected in the vicinity of the nucleus borders. The expression of TLR9 and TLR10 was moderate and granular throughout all nasal epithelial layers. The expression of MyD88 was patchy and varied from moderate to strong. Atopic female subjects had a lower staining score of TLR2, median (min–max) 0 (0–1); than atopic male subjects 1 1, 2, 3 (p = 0.04, by Fisher′s exact test, data not shown). There were no other gender‐ or age‐related differences in the baseline expression of TLR proteins (p > 0.05 by Fisher′s exact and Mann–Whitney *U*‐tests correspondingly, data not shown).

**Table 2:** Patient characteristics. Q1 and Q3 indicate 25% and 75% percentiles, respectively. p‐values by Fisher′s exact test (continuous variables); or by Kruskal–Wallis and Mann–Whitney *U*‐tests (dichotomous variables)

	Control	Atopy	p‐Value
	*N* = 10	*N* = 9	
Age			
Median	23	24	0.82
Min–max	22–36	22–34	
No. of male sex	3	3	1.00
Blood eosinophil count (10^9^/L)			
Median	0.09	0.13	0.66
Q1–Q3	0.08–0.19	0.10–0.24	
Serum total IgE (IU/mL)			
Median	27.0	98.0	0.013
Q1–Q3	11.5–72.0	57.0–694.0	
S‐IgE birch(IU/mL)			
Median	<0.35	12.0	0.001
Q1–Q3	<0.35–<0.35	8.0–192.5	
S‐IgE timothy grass(IU/mL)			
Median	<0.35	5.9	0.002
Q1–Q3	<0.35–<0.35	3.5–16.5	
No. subjects with positive SPT reaction to			
Any aeroallergen	0	9	<0.001
Birch pollen	0	9	<0.001
Timothy grass pollen	0	5	0.008
Other grass pollen	0	7	<0.001
Animal dander	0	5	0.008
House dust mite	0	0	1.00
Other aeroallergens	0	0	1.00
No. patients challenged with			
Diluent	4	3	1.00
Birch	6	6	
Itching symptom score median (min–max)			
Before challenge	0 (0–0)	0 (0–0)	1.00
After diluent	0 (0–0)	0 (0–0)	1.00
After birch	0 (0–0)	1 (0–2)	0.001
Congestion‐discharge‐sneezing score median (min–max)			
Before challenge	0 (0–0)	0 (0–0)	1.00
After diluent	0 (0–1)	1 (0–1)	0.49
After birch	0 (0–1)	0.5 (0–1)	1.00
Mucosal inflammation score median (min–max)			
Before challenge	1 (0–2)	1 (1–2)	1.00
After diluent	2 (1–2)	2 (1–2)	1.00
After birch	1 (0–2)	1.5 (1–2)	0.55

### The nasal epithelial expression of TLR proteins and genes after challenge

**Figure 3:**
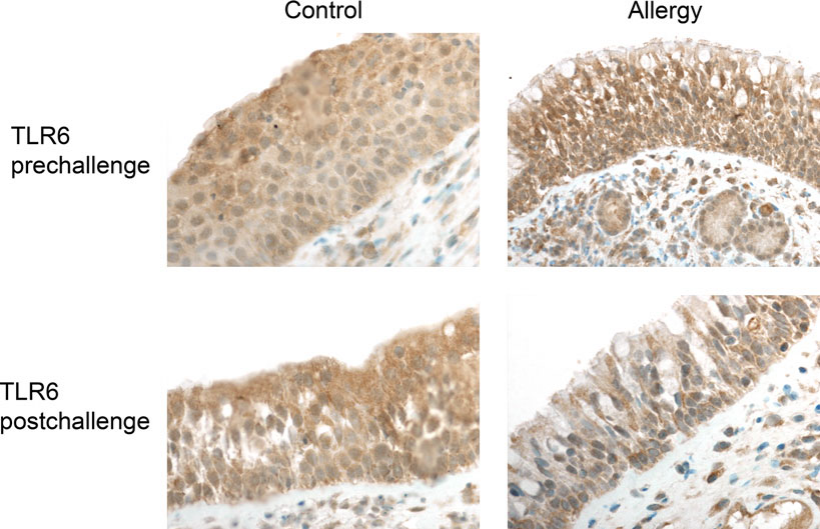
The expression of TLR6 in the nasal epithelium from a healthy subject (Control) and a birch pollen allergic (Allergy) subject in winter. The expression of TLR6 decreased significantly in the allergic group after the birch pollen challenge. Prechallenge – the nasal biopsy was taken before intranasal challenge with the birch pollen solution. Postchallenge – the nasal biopsy was taken 3 min after intranasal challenge with the birch pollen solution. Magnification ×200 in all panels.

After intranasal challenge with birch pollen solution, the median staining score of TLR6 was significantly lower in the atopic group contrasted to the healthy group (p = 0.02, Fig. 3), whereas no difference was observed after the diluent challenge. No differences were observed in the expression of TLR1‐5, TLR7, TLR9‐10, and MyD88 between the groups. There was no change in the median staining score of TLR1‐7 and 9‐10 proteins between nasal biopsies taken before and 3 min after the intranasal challenge in winter (p > 0.05, by Wilcoxon test, data not shown). The finding was similar in control and allergic groups, and after challenge with either birch pollen or diluent drops. The median delta staining scores of TLR1 and TLR6 were significantly lower in the atopic compared to the control group, but only after challenge with birch pollen (p = 0.04 both, Fig. 3). Interestingly, after the diluent challenge, the median delta staining score of TLR5 in the atopic group was 0 indicating no change, whereas in the control group it was 1.5 reflecting an increased TLR5 expression (p = 0.03 Fig. 3). The median delta staining scores of TLR2‐5, TLR7, TLR9‐10, and MyD88 did not differ between control and atopic groups, after challenge with birch pollen solution (p > 0.05, by Mann–Whitney *U*‐test, data not shown). After the challenge with a diluent solution, the median delta staining scores of TLR1‐4, TLR6‐7, TLR9‐10, and MyD88 did not differ between the control and atopic groups (p > 0.05, by Mann–Whitney *U*‐test, data not shown). When comparing nasal biopsies taken from the same individuals before and after the challenge with either diluent or birch pollen allergen drops the median staining scores of TLR 1‐7 and 9–10 proteins, and MyD88 did not differ (p > 0.05, by Wilcoxon test, data not shown).

**Figure 4:**
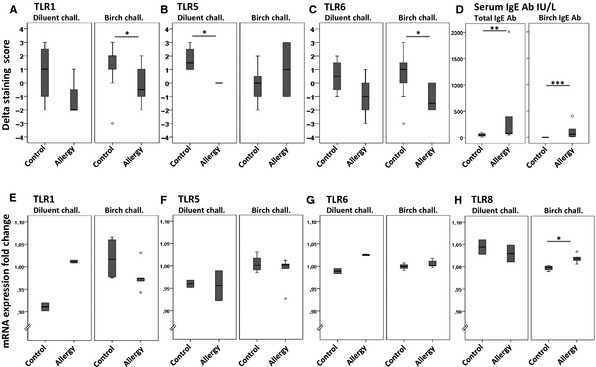
The immunohistochemical delta staining scores of TLR proteins (A–C) and TLR mRNA fold changes (E–H). Diluent chall. – intranasal challenge with diluent solution; Birch chall. – intranasal challenge with birch pollen solution. The staining score – proportion of positively stained cells/specimen (from 0 to 4). The delta staining score = staining score _postchallenge_ – staining score _prechallenge_. Only the TLR proteins with statistically significantly different delta staining scores between control (Control) and birch pollen allergic (Allergy) subjects are shown: TLR1 (A), TLR5 (B), and TLR6 (C). The mRNA fold change = relative mRNA abundancy _postchallenge_: relative mRNA abundancy _prechallenge_. The fold changes of TLR1 (E), TLR5 (F), TLR6 (G), and TLR8 (H) mRNAs. The levels of total and birch specific IgE Ab of serum. (D) *p < 0.05, **p < 0.01, ***p < 0.001, by Mann–Whitney *U*‐test.

The fold change values of TLR 1‐10 mRNAs ranged between 0.89 and 1.07, which indicates very slight changes on mRNA levels after challenge (Fig. 4). The median fold change of TLR8 mRNA was statistically significantly higher in allergic than control subjects after challenge with birch pollen, however the changes in TLR8 mRNA levels remained very slight and thus the difference was not biologically significant (Fig. 4H).

### Symptom scores and inflammation scores

In winter, the baseline median score for itching symptoms and for congestion‐discharge‐sneezing symptoms was identical in the control and atopic groups (p > 0.05 both, Table 2). Accordingly, the off‐seasonal baseline inflammation score of the biopsies was identical in both groups (p > 0.05 in both, Table 2). There were no change in the median inflammation scores when comparing specimens taken from the same individual before and 3 min after challenge with either diluent or birch, neither in controls nor in atopics (p > 0.05 all, by Wilcoxon test, data not shown). The median score for itching symptoms asked 20 min after birch challenge, was significantly higher in the atopic than in the control group, whereas no difference was detected after diluent challenge (p < 0.001, p = 1.00 correspondingly, Table 2). In contrast, all subjects reported postchallenge nasal pain/irritation, discharge, or slight bleeding, probably due to biopsy‐taking. Thus, there were no postchallenge differences between the atopic and control group in median score for congestion‐discharge‐sneezing symptoms (p > 0.05, Table 2). None of the subjects reported dyspnea after challenge.

## Discussion

In this study we showed that TLR 1‐7 and 9‐10 proteins are expressed in nasal epithelium. The TLR3, 6, 7, 10 proteins had the strongest nasal epithelial expression, whereas TLR2 had the mildest. TLR1, 2, 4, 5, 6, 10 proteins have previously been reported to be located in the plasma membrane; however we also detected them in the intracellular compartments of epithelial cells 8. Tengroth et al. demonstrated by RTq‐PCR, immunohistochemistry and flow cytometry that nasal apical epithelium expresses abundantly TLR3, TLR7, TLR9, RIG‐I, and MDA‐5. Moreover, they showed by ELISA upregulated cytokines (IL‐6, GMCSF, IL‐8, IFN‐b) in the nasal mucosa after stimulation of several TLR‐agonists, which suggests that epithelial TLR and RLR receptors might mediate nasal viral response and thus could be important in exacerbations 34. Bielinska et al. administered intranasally anthrax antigen with a nanoemulsion to mice. This induced TLR2 and TLR4 activation along with a MyD88‐independent antibody response and a MyD88‐dependent Th‐1 and Th‐17 cell‐mediated immune response 35. The finding might be used in the development of mucosal vaccines. Ioannidis et al. 36 demonstrated that TLR6 has a basolateral location in human tracheal epithelium which is in accordance to our observation in the nasal epithelium. TLR2‐6 proteins have been demonstrated to have the strongest expression in the airway epithelium while the expression of TLR7‐10 fluctuates depending on the cell type studied 5.

We previously demonstrated that birch pollen is actively transported through the epithelium within 1 min 31. In this study, we detected early changes in the expression of epithelial TLR1 or TLR6 after the birch pollen allergen challenge suggesting, that the challenge could putatively modify the structure of epithelial TLR1 or TLR6 proteins in atopic subjects leading to decreased binding of anti‐TLR1 and anti‐TLR6 antibodies to their epitopes. Thus, birch pollen allergen entry would putatively be regulated by TLR1 and TLR6 mediated pathways. The finding that mRNA expression was not affected after the challenge, could in part be explained by the fact that whole biopsies were used instead of epithelial cells. Our future aim would be to observe if birch exposure affected the activity of TLR proteins; or noncoding RNAs regulating TLR protein synthesis.

Fransson et al. demonstrated mRNA and protein expression of TLRs 2, 3, and 4 in the nasal mucosa both in subjects with and without birch and/or timothy pollen allergy, which is in accordance to our results 29. Probably partly due to differences in the study set up, they demonstrated a more apical location of TLR3 and an increase in expression of TLRs 2, 3, 4 proteins following the allergen challenge pre‐season, but an increase only in TLR3 mRNA during the pollen season 29.

Tengroth et al. have analyzed by flow cytometry and Luminex naïve nasal polyp and turbinate tissues, as well as human tissues after *in vivo* and *in vitro* stimulation with a TLR9 agonist, CpG. Interestingly, epithelial expression of TLR9 was detected in turbinates from healthy controls and in polyp tissue, whereas TLR9 was absent in turbinates from CRSwNP patients. CpG stimulation resulted in an upregulation of TLR9 and modulation of cytokines in turbinate tissue from patients, suggesting that defects in the TLR9 mediated microbial defense in the turbinate might explain virus‐induced polyp growth 37.

Several studies have observed TLRs in the atopic lower airway inflammation. TLR2‐TLR7 proteins have been detected in patients with severe asthma 38. Subcutaneous QbG10, a TLR9 agonist, improved symptoms and lung function in patients with allergic asthma 39. Another placebo‐controlled trial showed that repeated intranasal TLR7 agonist AZD8848 reduced symptoms in patients with birch and/or grass pollen allergic rhinitis, but it produced reversible blood lymphocyte reduction and dose‐dependent flu‐like symptoms as side‐effects 28. Intranasal TLR9 agonist CpG increased, whereas TLR7 agonist, R848, decreased airway inflammation in mice with established allergic inflammation 40. Parsons et al. 41 showed that primary bronchial epithelial cells from asthma patients were able to up‐regulate TLR3, following infection, but failed to initiate an effective innate immune response. Deifl et al. stimulated *in vitro* monocytes and monocyte‐derived dendritic cells from allergic patients with TLR ligands. They found that TLR ligands except flagellin enhanced Bet v 1 –allergen uptake 42. Our finding that the diluent challenge in controls induced expression of TLR5, which usually recognizes flagellin, requires further evidence to be explained.

In mice, allergic rhinitis response to house dust mite might result from TLR2 signaling axis in the nasal mucosa, whereas in the lung mucosa the allergic asthma response occurs predominantly via TLR4 signaling axis 43. Intranasally administered TLR3 or TLR4 ligands induced relatively similar murine airway hyper‐responsiveness and cellular infiltration in lungs 44. TLR2 agonist, Pam3Cys, induced an asthma exacerbation in mice, but had, on the other hand, long‐term protective effect on secondary allergic responses in the airways 45.

The TLR‐family with eleven members explodes in four‐figure numbers when observing single nucleotide polymorphism, expression variants, and post‐translational modification 46. Two meta‐analyses of genome‐wide association studies have shown that loci in TLR1‐TLR6‐TLR10 region associate with allergic sensitization; or self‐reported cat, dust mite, or pollen allergies 23, 24. Single nucleotide polymorphism in the TLR1, 6, 7, 8 genes might associate with grass and/or birch pollen allergic rhinitis; of which the association between TLR7‐8 gene variants and grass pollen allergic rhinitis was more pronounced 26, 27. Other studies have shown that polymorphisms or defects in TLR1‐2, TLR6‐7, TLR9‐10 genes, and MyD‐88 dependent pathways seem to associate with atopic asthma 13, 47, 48, 49. Our findings of epithelial TLR1‐2, TLR6, TLR9, and MyD88 having putatively a role in birch pollen allergic responses is consistent with these observations. Future evidence is still needed of gene‐environmental interactions affecting the TLR pathways in airway allergy.

Our results should be interpreted with caution because of the small number of patients studied. The usage of the whole biopsies and short challenge time, might have in part affected the results. We also acknowledge the fact that the effect of multiple testing could limit the significance of the results.

## Conclusions

The TLR protein family is widely located in the human nasal epithelium, which most probably reflects to the active innate immunity functions of nasal epithelium. Findings of altered TLR1 and TLR6 protein expression after birch pollen challenge still require to be validated on the protein activity level.

## Conflict of Interests

The authors declare that they have no conflict of interests.

## Authors' Contributions

Sanna Toppila‐Salmi and Mikko Lehtonen recruited the volunteers and performed the sampling. Jutta Renkonen, Jaana Hagström, Caj Haglund, and ST‐S were responsible for immunohistochemistry and microscopy. Sakari Joenväärä, Pirkko Mattila and Ville Parviainen performed the laboratory experiments and analyses. Risto Renkonen, JR and ST‐S provided the study plan, data analyses and wrote the manuscript. All authors reviewed critically the manuscript.

The study was supported in part by research grants from Academy of Finland, Sigrid Juselius Foundation, and Helsinki University Central Hospital Research Funds, Competitive Research Funding of the Tampere University Hospital (Grant 9L087), the Finnish Society of Allergology and Immunology, the Finnish Medical Society Duodecim, the Väinö and Laina Kivi Foundation, the Yrjö Jahnsson Foundation, and Ahokas Foundation. The authors thank Raija Hukkila, Marja‐Leena Koskinen, Satu Lehti, Marja‐Leena Oksanen, and Päivi Peltokangas for excellent technical assistance. The volunteers are warmly thanked for participation in this study.

## References

[1] Bousquet J , Schunemann HJ , Samolinski B , Demoly P , Baena‐Cagnani CE , Bachert C , et al. Allergic Rhinitis and its Impact on Asthma (ARIA): achievements in 10 years and future needs. J Allergy Clin Immunol 2012;130:1049–62. 23040884 10.1016/j.jaci.2012.07.053

[2] Haahtela T , von Hertzen L , Makela M , Hannuksela M , Allergy Programme Working G . Finnish Allergy Programme 2008‐2018–time to act and change the course. Allergy 2008;63:634–45. 18445181 10.1111/j.1398-9995.2008.01712.x

[3] Golebski K , Roschmann KI , Toppila‐Salmi S , Hammad H , Lambrecht BN , Renkonen R , et al. The multi‐faceted role of allergen exposure to the local airway mucosa. Allergy 2013;68:152–60. 23240614 10.1111/all.12080

[4] Holgate ST . Mechanisms of asthma and implications for its prevention and treatment: a personal journey. Allergy Asthma Immunol Res 2013;5:343–7. 24179679 10.4168/aair.2013.5.6.343 PMC3810539

[5] Gras D , Chanez P , Vachier I , Petit A , Bourdin A . Bronchial epithelium as a target for innovative treatments in asthma. Pharmacol Ther 2013;140:290–305. 23880290 10.1016/j.pharmthera.2013.07.008

[6] Jacquet A . The role of innate immunity activation in house dust mite allergy. Trends Mol Med 2011;17:604–11. 21741880 10.1016/j.molmed.2011.05.014

[7] Bauer S , Muller T , Hamm S . Pattern recognition by Toll‐like receptors. Adv Exp Med Biol 2009;653:15–34. 19799109 10.1007/978-1-4419-0901-5_2

[8] Moresco EM , LaVine D , Beutler B . Toll‐like receptors. Curr Biol 2011;21:R488–93. 21741580 10.1016/j.cub.2011.05.039

[9] Gribar SC , Richardson WM , Sodhi CP , Hackam DJ . No longer an innocent bystander: epithelial toll‐like receptor signaling in the development of mucosal inflammation. Mol Med 2008;14:645–59. 18584047 10.2119/2008-00035.Gribar PMC2435494

[10] Wells JM , Rossi O , Meijerink M , van Baarlen P . Epithelial crosstalk at the microbiota‐mucosal interface. Proc Natl Acad Sci USA 2011;108(Suppl 1):4607–14. 20826446 10.1073/pnas.1000092107 PMC3063605

[11] Chen K , Xiang Y , Yao X , Liu Y , Gong W , Yoshimura T , et al. The active contribution of Toll‐like receptors to allergic airway inflammation. Int Immunopharmacol 2011;11:1391–8. 21624504 10.1016/j.intimp.2011.05.003 PMC7398422

[12] Lin CF , Tsai CH , Cheng CH , Chen YS , Tournier F , Yeh TH . Expression of Toll‐like receptors in cultured nasal epithelial cells. Acta Otolaryngol 2007;127:395–402. 17453460 10.1080/00016480601089416

[13] Tesse R , Pandey RC , Kabesch M . Genetic variations in toll‐like receptor pathway genes influence asthma and atopy. Allergy 2011;66:307–16. 21039600 10.1111/j.1398-9995.2010.02489.x

[14] Vandenbon A , Teraguchi S , Akira S , Takeda K , Standley DM . Systems biology approaches to toll‐like receptor signaling. Wiley Interdiscip Rev Syst Biol Med 2012;4:497–507. 22714995 10.1002/wsbm.1178 PMC3465798

[15] Kubinak JL , Round JL . Toll‐like receptors promote mutually beneficial commensal‐host interactions. PLoS Pathog 2012;8:e1002785. 22910541 10.1371/journal.ppat.1002785 PMC3406078

[16] Parsons KS , Hsu AC , Wark PA . TLR3 and MDA5 signalling though not expression, is impaired in asthmatic epithelial cells in response to rhinovirus infection. Clin Exp Allergy 2014;44:91–101. 24131248 10.1111/cea.12218

[17] Ray A , Chakraborty K , Ray P . Immunosuppressive MDSCs induced by TLR signaling during infection and role in resolution of inflammation. Front Cell Infect Microbiol 2013;3:52. 24066282 10.3389/fcimb.2013.00052 PMC3776133

[18] Habibzay M , Saldana JI , Goulding J , Lloyd CM , Hussell T . Altered regulation of Toll‐like receptor responses impairs antibacterial immunity in the allergic lung. Mucosal Immunol 2012;5:524–34. 22549744 10.1038/mi.2012.28 PMC3427016

[19] Li C , Shi L , Yan Y , Gordon BR , Gordon WM , Wang DY . Gene expression signatures: a new approach to understanding the pathophysiology of chronic rhinosinusitis. Curr Allergy Asthma Rep 2013;13:209–17. 23225138 10.1007/s11882-012-0328-6

[20] Cario E . Toll‐like receptors in inflammatory bowel diseases: a decade later. Inflamm Bowel Dis 2010;16:1583–97. 20803699 10.1002/ibd.21282 PMC2958454

[21] Cole JE , Georgiou E , Monaco C . The expression and functions of toll‐like receptors in atherosclerosis. Mediators Inflamm 2010;2010:393946. 20652007 10.1155/2010/393946 PMC2905957

[22] Jin C , Flavell RA . Innate sensors of pathogen and stress: linking inflammation to obesity. J Allergy Clin Immunol 2013;132:287–94. 23905917 10.1016/j.jaci.2013.06.022

[23] Bonnelykke K , Matheson MC , Pers TH , Granell R , Strachan DP , Alves AC , et al. Meta‐analysis of genome‐wide association studies identifies ten loci influencing allergic sensitization. Nat Genet 2013;45:902–6. 23817571 10.1038/ng.2694 PMC4922420

[24] Hinds DA , McMahon G , Kiefer AK , Do CB , Eriksson N , Evans DM , et al. A genome‐wide association meta‐analysis of self‐reported allergy identifies shared and allergy‐specific susceptibility loci. Nat Genet 2013;45:907–11. 23817569 10.1038/ng.2686 PMC3753407

[25] Hammad H , Chieppa M , Perros F , Willart MA , Germain RN , Lambrecht BN . House dust mite allergen induces asthma via Toll‐like receptor 4 triggering of airway structural cells. Nat Med 2009;15:410–6. 19330007 10.1038/nm.1946 PMC2789255

[26] Moller‐Larsen S , Nyegaard M , Haagerup A , Vestbo J , Kruse TA , Borglum AD . Association analysis identifies TLR7 and TLR8 as novel risk genes in asthma and related disorders. Thorax 2008;63:1064–9. 18682521 10.1136/thx.2007.094128

[27] Nilsson D , Andiappan AK , Hallden C , De Yun W , Sall T , Tim CF , et al. Toll‐like receptor gene polymorphisms are associated with allergic rhinitis: a case control study. BMC Med Genet 2012;13:66. 22857391 10.1186/1471-2350-13-66 PMC3459792

[28] Greiff L , Cervin A , Ahlstrom‐Emanuelsson C , Almqvist G , Andersson M , Dolata J , et al. Repeated intranasal TLR7 stimulation reduces allergen responsiveness in allergic rhinitis. Respir Res 2012;13:53. 22726593 10.1186/1465-9921-13-53 PMC3487914

[29] Fransson M , Adner M , Erjefalt J , Jansson L , Uddman R , Cardell LO . Up‐regulation of Toll‐like receptors 2, 3 and 4 in allergic rhinitis. Respir Res 2005;6:100. 16146574 10.1186/1465-9921-6-100 PMC1243240

[30] Mattila P , Renkonen J , Toppila‐Salmi S , Parviainen V , Joenvaara S , Alff‐Tuomala S , et al. Time‐series nasal epithelial transcriptomics during natural pollen exposure in healthy subjects and allergic patients. Allergy 2010;65:175–83. 19804444 10.1111/j.1398-9995.2009.02181.x

[31] Joenvaara S , Mattila P , Renkonen J , Makitie A , Toppila‐Salmi S , Lehtonen M , et al. Caveolar transport through nasal epithelium of birch pollen allergen Bet v 1 in allergic patients. J Allergy Clin Immunol 2009;124:135–42.e1‐21. 19344938 10.1016/j.jaci.2008.11.048

[32] Luukkainen A , Karjalainen J , Honkanen T , Lehtonen M , Paavonen T , Toppila‐Salmi S . Indoleamine 2,3‐dioxygenase expression in patients with allergic rhinitis: a case‐control study. Clin Transl Allergy 2011;1:17‐7022‐1‐17. 10.1186/2045-7022-1-17 PMC3299587 22410120

[33] Kallio MA , Tuimala JT , Hupponen T , Klemela P , Gentile M , Scheinin I , et al. Chipster: user‐friendly analysis software for microarray and other high‐throughput data. BMC Genom 2011;12:507‐2164‐12‐507. 10.1186/1471-2164-12-507 PMC3215701 21999641

[34] Tengroth L , Millrud CR , Kvarnhammar AM , Kumlien Georen S , Latif L , Cardell LO . Functional effects of Toll‐like receptor (TLR)3, 7, 9, RIG‐I and MDA‐5 stimulation in nasal epithelial cells. PLoS ONE 2014;9:e98239. 24886842 10.1371/journal.pone.0098239 PMC4041746

[35] Bielinska AU , Makidon PE , Janczak KW , Blanco LP , Swanson B , Smith DM , et al. Distinct pathways of humoral and cellular immunity induced with the mucosal administration of a nanoemulsion adjuvant. J Immunol 2014;192:2722–33. 24532579 10.4049/jimmunol.1301424 PMC3948110

[36] Ioannidis I , Ye F , McNally B , Willette M , Flano E . Toll‐like receptor expression and induction of type I and type III interferons in primary airway epithelial cells. J Virol 2013;87:3261–70. 23302870 10.1128/JVI.01956-12 PMC3592129

[37] Tengroth L , Arebro J , Kumlien Georen S , Winqvist O , Cardell LO . Deprived TLR9 expression in apparently healthy nasal mucosa might trigger polyp‐growth in chronic rhinosinusitis patients. PLoS ONE 2014;9:e105618. 25133733 10.1371/journal.pone.0105618 PMC4136868

[38] Moreira AP , Cavassani KA , Ismailoglu UB , Hullinger R , Dunleavy MP , Knight DA , et al. The protective role of TLR6 in a mouse model of asthma is mediated by IL‐23 and IL‐17A. J Clin Invest 2011;121:4420–32. 22005301 10.1172/JCI44999 PMC3204826

[39] Beeh KM , Kanniess F , Wagner F , Schilder C , Naudts I , Hammann‐Haenni A , et al. The novel TLR‐9 agonist QbG10 shows clinical efficacy in persistent allergic asthma. J Allergy Clin Immunol 2013;131:866–74. 23384679 10.1016/j.jaci.2012.12.1561

[40] Adner M , Starkhammar M , Georen SK , Dahlen SE , Cardell LO . Toll‐like receptor (TLR) 7 decreases and TLR9 increases the airway responses in mice with established allergic inflammation. Eur J Pharmacol 2013;718:544–51. 24041926 10.1016/j.ejphar.2013.09.004

[41] Parsons KS , Hsu AC , Wark PA . TLR3 and MDA5 signalling, although not expression, is impaired in asthmatic epithelial cells in response to rhinovirus infection. Clin Exp Allergy 2014;44:91–101. 24131248 10.1111/cea.12218

[42] Deifl S , Kitzmuller C , Steinberger P , Himly M , Jahn‐Schmid B , Fischer GF , et al. Differential activation of dendritic cells by toll‐like receptors causes diverse differentiation of naive CD4 T cells from allergic patients. Allergy 2014;69:1602–9. 25093709 10.1111/all.12501 PMC4245478

[43] Ryu JH , Yoo JY , Kim MJ , Hwang SG , Ahn KC , Ryu JC , et al. Distinct TLR‐mediated pathways regulate house dust mite‐induced allergic disease in the upper and lower airways. J Allergy Clin Immunol 2013;131:549–61. 23036747 10.1016/j.jaci.2012.07.050

[44] Starkhammar M , Kumlien Georen S , Swedin L , Dahlen SE , Adner M , Cardell LO . Intranasal administration of poly(I:C) and LPS in BALB/c mice induces airway hyperresponsiveness and inflammation via different pathways. PLoS ONE 2012;7:e32110. 22355412 10.1371/journal.pone.0032110 PMC3280225

[45] Nawijn MC , Motta AC , Gras R , Shirinbak S , Maazi H , van Oosterhout AJ . TLR‐2 activation induces regulatory T cells and long‐term suppression of asthma manifestations in mice. PLoS ONE 2013;8:e55307. 23393567 10.1371/journal.pone.0055307 PMC3564817

[46] Renkonen J , Mattila P , Parviainen V , Joenvaara S , Toppila‐Salmi S , Renkonen R . A network analysis of the single nucleotide polymorphisms in acute allergic diseases. Allergy 2010;65:40–7. 19796227 10.1111/j.1398-9995.2009.02101.x

[47] Kormann MS , Depner M , Hartl D , Klopp N , Illig T , Adamski J , et al. Toll‐like receptor heterodimer variants protect from childhood asthma. J Allergy Clin Immunol 2008;122:86–92, 92.e1‐8. 18547625 10.1016/j.jaci.2008.04.039

[48] Klaassen EM , Thonissen BE , van Eys G , Dompeling E , Jobsis Q . A systematic review of CD14 and toll‐like receptors in relation to asthma in Caucasian children. Allergy Asthma Clin Immunol 2013;9:10‐1492‐9‐10. 10.1186/1710-1492-9-10 PMC3602113 23496969

[49] Kaiko GE , Loh Z , Spann K , Lynch JP , Lalwani A , Zheng Z , et al. Toll‐like receptor 7 gene deficiency and early‐life Pneumovirus infection interact to predispose toward the development of asthma‐like pathology in mice. J Allergy Clin Immunol 2013;131:1331–9.e10. 23561801 10.1016/j.jaci.2013.02.041

